# Supplementary material for: Segmented SiPM Readout for Cherenkov Time-of-Flight Positron Emission Tomography Detectors Based on Bismuth Germanate
Source: ACS Photonics. 2025 Feb 6;12(2):1125–36. doi: 10.1021/acsphotonics.4c02265 (PMC11843713; doi:10.1021/acsphotonics.4c02265)
Supplement: Supplementary file 1 — ph4c02265_si_001.pdf [file ph4c02265_si_001.pdf]

Supporting Information

Segmented readout for Cherenkov time-of-flight positron emission tomography detectors based on bismuth germanate

Minseok Yi[1,2,3], Daehee Lee[1], Alberto Gola[4], Stefano Merzi[4], Michele Penna[4], Jae Sung Lee[2,3,5], Simon R. Cherry[1], Sun Il Kwon[1, *]

[1]*Department of Biomedical Engineering, University of California, Davis, Davis, California 95616, United States*

[2]*Interdisciplinary Program in Bioengineering, Seoul National University College of Engineering, Seoul 03080, Republic of Korea*

[3]*Integrated Major in Innovative Medical Science, Seoul National University, Seoul 03080, Republic of Korea*

[4]*Fondazione Bruno Kessler, Trento I-38123, Italy*

[5]*Brightonix Imaging Inc., Seoul 04782, Republic of Korea*

*Corresponding author: sunkwon@ucdavis.edu

Table S1. Coincidence timing resolution values measured using different BGO crystal lengths.

Length	5 mm		10 mm		15 mm		20 mm	
[ps]	FWHM	FWTM	FWHM	FWTM	FWHM	FWTM	FWHM	FWTM
T_A	185 ± 3	826 ± 36	210 ± 11	1084 ± 56	230 ± 12	1200 ± 70	255 ± 14	1257 ± 53
T_B	189 ± 4	861 ± 43	203 ± 13	1005 ± 22	224 ± 8	1131 ± 45	250 ± 13	1277 ± 44
T_{Early} All Events	167 ± 3	423 ± 27	175 ± 5	502 ± 17	186 ± 5	590 ± 22	204 ± 5	648 ± 28
T_{Early} ($\Delta T_k = 300\ ps$)	161 ± 6	359 ± 11	164 ± 4	399 ± 16	183 ± 8	500 ± 28	181 ± 10	500 ± 20

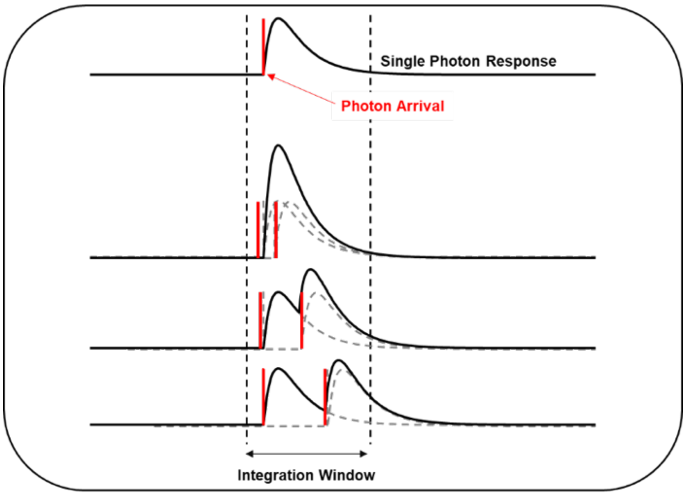

Figure S1. The effect of photon arrival delay on the integration value.

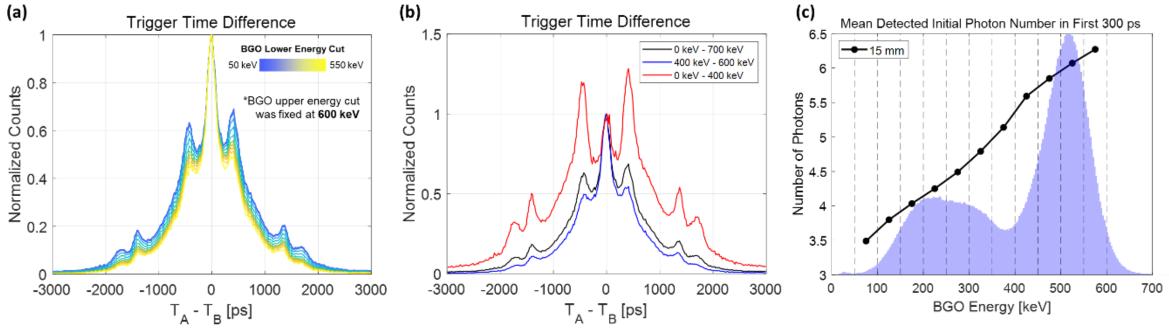

Figure S2. Distributions of trigger time differences with varying energy lower limits. (a) With a fixed 600 keV upper limit (b) Photopeak (400 – 700 keV) and scatter (0 – 400 keV) energy regions. (c) The number of initial photons for different energy intervals (50 – 100 keV, 100 – 150 keV, …, 550 – 600 keV).

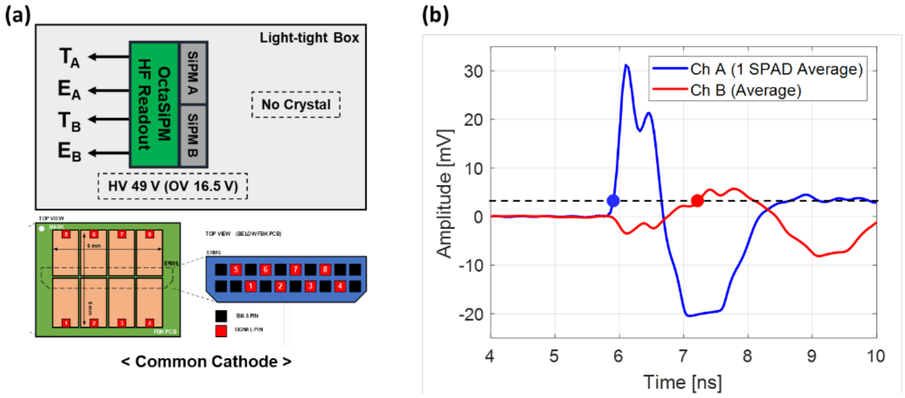

Figure S3. Electronic crosstalk due to the common cathode configuration.

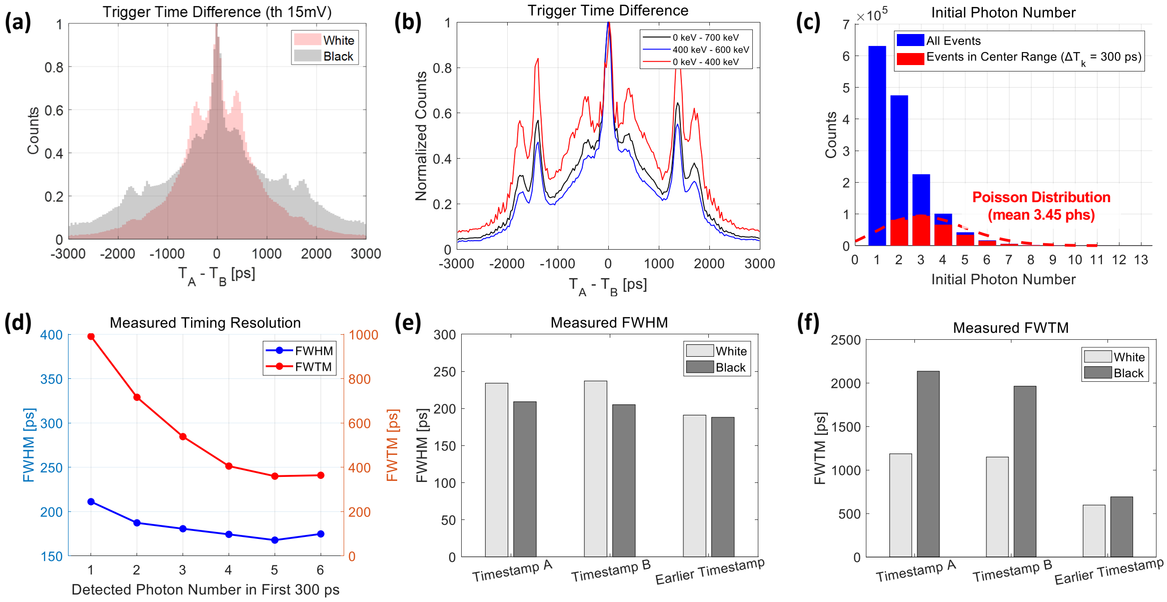

Figure S4. Black reflector results: (a) trigger time difference histograms for BGO pixels wrapped with white and black-painted PTFE tape, respectively. (b) distributions of trigger time differences for different energy ranges. (c) distributions of the initial photon number for different trigger time difference ranges. (d) measured FWHM and FWTM values. Measured FWHM (e) and FWTM (f) using different timestamps.

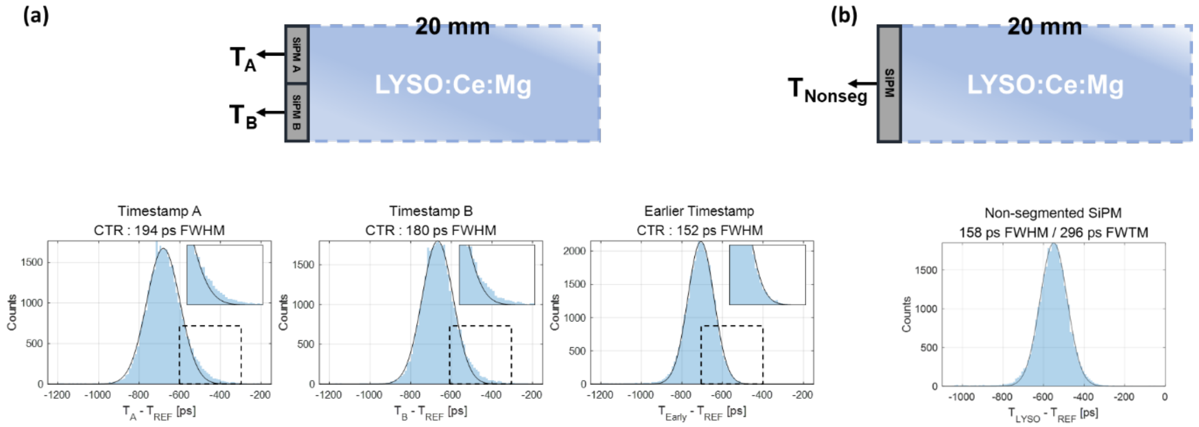

Figure S5. Measurements from LYSO:(Ce,Mg) coupled to an OctaSiPM (a) and a non-segmented SiPM (b).
